# Angiotensin AT_1_ – α_2C_-Adrenoceptor Interaction Disturbs α_2A_-auto-Inhibition of Catecholamine Release in Hypertensive Rats

**DOI:** 10.3389/fneur.2013.00070

**Published:** 2013-06-10

**Authors:** Torill Berg

**Affiliations:** ^1^Department of Physiology, Institute of Basic Medical Sciences, University of Oslo, Oslo, Norway

**Keywords:** α_2_-adrenoceptors, angiotensin AT1 receptor, sympathetic nervous system, norepinephrine, epinephrine, release-control, spontaneously hypertensive rats, total peripheral vascular resistance

## Abstract

α_2_-Adrenoceptors lower central sympathetic output and peripheral catecholamine release, and thus may prevent sympathetic hyperactivity and hypertension. α_2_AR also influence vascular tension. These α_2_AR are malfunctioning in spontaneously hypertensive rats (SHR). Here I tested if an interaction between α_2_AR subtypes and the angiotensin AT1 receptor (AT_1_R) precipitated these disorders. Blood pressure was monitored through a femoral artery catheter and cardiac output by ascending aorta flow in anesthetized rats. Catecholamine concentrations were determined in plasma collected at the end of a 15-min tyramine-infusion. Tyramine stimulates norepinephrine release through the re-uptake transporter, thus preventing re-uptake. Presynaptic control of vesicular release is therefore reflected as differences in overflow to plasma. Previous experiments showed surgical stress to activate some secretion of epinephrine, also subjected to α_2_AR-auto-inhibition. Normotensive rats (WKY) and SHR were pre-treated with (1) vehicle or α_2_AR-antagonist (L-659,066), followed by fadolmidine (α_2C>B>A_ + α_1_AR-agonist), ST-91 (α_2non-A_-selective agonist), or *m*-nitrobiphenyline (α_2C_AR-agonist + α_2A+B_-antagonist), or (2) AT1R-antagonist losartan, losartan + L-659,066, or losartan + clonidine. In WKY, L-659,066 alone, L-659,066 + agonist or losartan + L-659,066 increased catecholamine overflow to plasma after tyramine and eliminated the norepinephrine-induced rise in total peripheral vascular resistance (TPR). In SHR, L-659,066 + fadolmidine/ST-91/*m*-nitrobiphenyline and losartan + L-659,066 greatly increased, and losartan + clonidine reduced, catecholamine concentrations, and L-659,066 + ST-91, losartan + L-659,066 and losartan + clonidine eliminated the tyramine-induced rise in TPR. Separately, these drugs had no effect in SHR. In conclusion, peripheral α_2C_AR-stimulation or AT_1_R-inhibition restored failing α_2A_AR-mediated auto-inhibition of norepinephrine and epinephrine release and control of TPR in SHR.

## Introduction

Sympathetic hyperactivity is a major force in initiating and sustaining spontaneous hypertension (Guyenet, 2006; Esler, 2011). α_2_-adrenoceptors (AR) lower sympathetic output from the central nervous system (CNS), and inhibit release of norepinephrine from peripheral sympathetic nerves and catecholamines from the adrenal medulla (Starke, 2001). Their activation is tonic, and they hamper release even in the anesthetized rat without stimulation of norepinephrine release (Berg et al., 2012). They therefore represent the last line of defense against sympathetic hyperactivity, and, if not functioning, plasma norepinephrine levels and blood pressure (BP) will increase, as demonstrated in genetically modified mice (Makaritsis et al., 1999). In the spontaneously hypertensive rat (SHR), deficiencies have been detected in both central and peripheral α_2_AR-mediated inhibition of release (Remie et al., 1992; Zugck et al., 2003). We have recently demonstrated that during tyramine-stimulated norepinephrine release, α_2_AR failed to lower norepinephrine and epinephrine release in SHR, and also failed to control vascular tension (Berg and Jensen, 2013). These malfunctions were not detected without activation of norepinephrine release (Berg et al., 2012), indicating that they resulted from the released catecholamine itself, or another agent released by, or co-released with norepinephrine or epinephrine. Surprisingly, these peripheral disorders were repaired by the non-selective agonist clonidine, which reduced catecholamine release, and also, through a central action, normalized the high resting BP, heart rate (HR), and total peripheral vascular resistance (TPR) in SHR (Berg et al., 2012).

**Figure 1:**
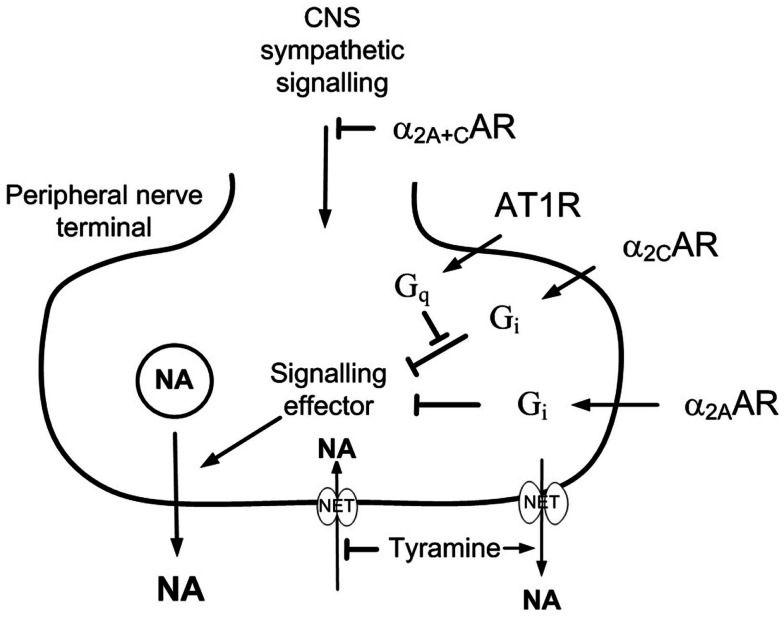
**The effect of presynaptic α_2C_AR and AT_1_R on norepinephrine release**. AT_1_R-G_q_-signaling stimulates norepinephrine release by interfering with the down-stream signaling of G_i_ (Cox et al., 2000). The AT_1_R/α_2_AR interaction involved only the α_2C_- and not the α_2A_-subtype (Trendelenburg et al., 2003a). The present results show that α_2C_AR-stimulation or AT_1_R-inhibition was required for α_2A_AR to effectively moderate peripheral norepinephrine release in SHR during tyramine-stimulated norepinephrine release. This malfunction may be due to excessive AT_1_R-G_q_-signaling in this strain, and α_2A_-signaling was evidently not permitted as long as AT_1_R-G_q_-signaling interfered with the function of the α_2C_AR. Tyramine stimulates reverse transport of norepinephrine through NET, and therefore also prevents synaptic NET re-uptake, allowing presynaptic control of release to be reflected as differences in overflow and the plasma norepinephrine concentration.

The restoring effect of clonidine may result from its central action or from an interaction between presynaptic receptors. α_2_AR are divided into three subtypes, i.e., α_2A_, α_2B_, and α_2C_. The α_2A_-and α_2C_-subtypes mediated the inhibition of central sympathetic output, whereas all three subtypes may reduce norepinephrine release from peripheral sympathetic nerves (Hein et al., 1999; Trendelenburg et al., 2003b) and the adrenal medulla (Brede et al., 2003; Moura et al., 2006). Inhibition of adrenal epinephrine release involved the α_2C_-subtype in the mouse (Brede et al., 2003; Moura et al., 2006), but the α_2A_-subtype in rat and man (Lymperopoulos et al., 2007; Berg et al., 2012). It has been shown that on-going α_2_AR-signaling markedly enhanced the stimulating effect of the angiotensin AT1 receptor (AT_1_R) – phospholipase C – protein kinase C (PKC) pathway on norepinephrine release in the rat vas deferens (Talaia et al., 2006). Similarly, studies on tissues from genetically modified mice (Trendelenburg et al., 2003a) demonstrated that the enhancing effect of release-stimulating receptors, including the AT_1_R, depended on active α_2_AR-signaling. However, the interaction involved the α_2C_AR-subtype only (Figure 1). Since the renin angiotensin system plays a significant role in hypertension pathology in SHR, I hypothesized that the clonidine-dependent restoration of α_2_AR inhibition of release in SHR involved stimulation of the α_2C_AR, thus counter-acting an excessive AT_1_R-signaling.

The angiotensin II responsible for a possible AT_1_R interference in SHR is not likely to origin from the sympathetic nerves themselves. Therefore, to have all components present, a role of the AT_1_R in the α_2_AR malfunction in SHR should be tested *in vivo*, which represents an experimental challenge. Due to synaptic uptake of norepinephrine through the norepinephrine re-uptake transporter (NET), presynaptic modulation of release is not reflected as differences in overflow and the plasma norepinephrine concentration (Berg et al., 2012). However, when NET-mediated re-uptake was blocked by desipramine, α_2_AR-antagonists greatly increased the plasma concentration of norepinephrine in the resting, anesthetized rat, in which norepinephrine release was not stimulated. Overflow to plasma under resting conditions is low, and inhibition of release by α_2_AR-agonist had no or little effect on the plasma norepinephrine concentration (Berg et al., 2012). In addition, the α_2_AR malfunction in SHR was not observed unless norepinephrine release was activated. Peripheral norepinephrine release can be stimulated by tyramine, which activates reverse transport through NET. Most likely by engaging NET in release, thus preventing re-uptake, presynaptic α_2_AR modulation altered tyramine-induced norepinephrine overflow to plasma, similar to that after desipramine in not-stimulated rats (Berg and Jensen, 2013). Restored α_2_AR control of release after α_2C_AR-stimulation or AT_1_R-antagonist could therefore be tested by the ability of the non-selective α_2_AR-antagonist L-659,066 to increase tyramine-induced norepinephrine overflow to plasma.

Epinephrine released in the adrenals is not subjected to re-uptake, and is not stimulated by tyramine. However, the stress induced by the surgical procedure activated some secretion of epinephrine, which was also subjected to α_2_AR-mediated release-control (Berg et al., 2012; Berg and Jensen, 2013).

Due to the activation of norepinephrine release, tyramine in addition induced a sympathetic cardiovascular response. This response was not influenced by baroreceptor activation, demonstrated by that baroreceptor control of HR was abolished by the pentobarbital-anesthesia (Berg et al., 2012). Moreover, epinephrine secretion is not regulated by the baroreceptor reflex. Thus, by recording BP and cardiac output (CO), the implications of altered catecholamine release and a possible postsynaptic α_2_AR/AT_1_R interaction in the control of TPR could be evaluated.

The results will show that the failing α_2_AR control of norepinephrine and epinephrine release and modulation of the norepinephrine-induced rise in TPR in SHR was restored by stimulation of peripheral α_2C_AR or inhibition of the AT_1_R.

## Materials and Methods

### Experimental procedure

All experiments were approved by the institutional review committee, and conducted in accordance with the Directive 2010/63/EU of the European Parliament. About 12–14 weeks old, male normotensive rats (Wistar Kyoto, WKY, *n* = 99, 284 ± 3 g b.w.) and SHR (Okamoto, SHR/NHsd strain, *n* = 107, 288 ± 2 g b.w.) on 12/12 h light/dark cycles were allowed conventional rat chow diet (0.7% NaCl) and water *ad lib* until the time of the experiment. The rats were anesthetized with pentobarbital (70–75 mg/kg, i.p.). As previously described (Berg et al., 2010; Berg and Jensen, 2013), mean arterial BP [MBP = (systolic BP − diastolic BP)/3 + diastolic BP] was monitored through a catheter in the femoral artery, flushed with 0.15 ml PBS (0.01 M Na-phosphate, 0.14 M NaCl, pH 7.4) containing 500 IU heparin/ml. CO and HR were recorded by a flow probe on the ascending aorta. TPR (MBP/CO) was calculated. The rats were on a positive-pressure ventilator throughout the experiment, ventilated with air. Previous measurements of blood gas parameters demonstrated adequate ventilation in both strains (Berg, 2002, 2003). Positive-pressure ventilation reduces right atrium ejection, and consequently lowered CO and MBP. This reduction was significant in SHR, but did not appear to influence the stimulated adrenergic responses, as previously discussed (Berg and Jensen, 2013). Body temperature was maintained at 37–38 °C by external heating, guided by a thermo sensor, inserted inguinally into the abdominal cavity.

### Experimental design

Control rats were pre-treated with PBS and infused for 15 min with tyramine to induce NET-mediated norepinephrine release. Since subtype-selective α_2_AR-agonists, which do not cross the blood-brain barrier, are not available, I used α_2_AR-agonists with different subtype profiles and different ability to cross the blood-brain barrier. Rats were therefore pre-treated with PBS or the α_2_AR-antagonist L-659,066, followed 10 min later by α_2_AR-agonist, i.e., fadolmidine, ST-91, or (R)-(+)-*m*-nitrobiphenyline oxalate. Rats were also pre-treated with the AT_1_R-antagonist losartan, alone or followed by L-659,066, clonidine, or ST-91. Drug specificity and dose are given in Table 1. Blood for the measurement of catecholamines was collected from the arterial catheter after the 15-min tyramine-observation period, but without discontinuing the infusion.

**Table 1:** **Mode of action and dose of the pharmacological agents used**.

Drug	Mode of action	Crosses blood-brain barrier	Dose per kg
Tyramine	Norepinephrine efflux through NET	No	1.26 μmol/min (Berg et al., 2010)
Clonidine	α_2_AR-agonist (non-selective)	Yes	151 nmol (Berg et al., 2012; Berg and Jensen, 2013)
Fadolmidine (Lehtimaki et al., 2008)	α_2C>B>A_AR-agonist (+α_1_AR-agonist activity)	No	2 nmol^a^
ST-91 (Takano et al., 1992)	α_2_AR-agonist (non-α_2A_)	No	24 nmol^a^
*m*-nitrobiphenyline (Crassous et al., 2007)	α_2_AR-agonist (α_2C_-selective) (+α_2A+B_AR-antagonist activity)	Not known	12.4 nmol^a^
L-659,066 (Clineschmidt et al., 1988)	α_2_AR-antagonist (non-selective)	No	4.4 μmol (Berg et al., 2012; Berg and Jensen, 2013)
Losartan	AT_1_R-antagonist	Yes (Li et al., 1993)	79 μmol (Berg, 2002)

*^a^Concentration established in preliminary tests to give a substantial (50–100%) but sub-maximal increase in MBP. Tyramine was administered as a 15-min infusion, whereas the other drugs were administered as bolus injections (0.6–1.0 ml/kg) 10 min before tyramine, except clonidine, which was injected 15 min before. All drugs were dissolved in PBS, and administered through a catheter in the femoral vein. When pre-treatment consisted of two drugs, these were given 10 min apart*.

### Measurement of plasma catecholamines

About 1.5 ml blood was collected into tubes containing 40 μl 0.2 M glutathione and 0.2 M EGTA (4 °C). Plasma was stored at −80 °C until the norepinephrine and epinephrine concentrations were determined, using 400 μl plasma and the 5000 Reagent kit for HPLC analysis of Catecholamines in plasma from Chromsystems GmbH, Munich, Germany, as described by the manufacturer.

### Drugs

Pentobarbital was from the Norwegian National Hospital, Oslo, Norway. L-659,066 was a kind gift from Merck, Sharp, and Dohme Labs, Rahway, NJ, USA, and fadolmidine HCl from Orion Corporation, Espoo, Finland. ST-91 was from TOCRIS bioscience, Bristol, UK; and (R)-(+)-*m*-nitrobiphenyline oxalate from Santa Cruz Biotechnology, Heidelberg, Germany. The remaining drugs were from Sigma Chemical Co., St. Louis, MO, USA.

### Statistical analyses

Results are presented as mean values ± SEM. Changes in the cardiovascular parameters were expressed in % of baseline. Data were averaged every min in all experiments. For the narrow peak-pressor response to ST-91 and *m*-nitrobiphenyline, data were averaged every 5 s. The cardiovascular response-curves to agonists and tyramine were analyzed using Repeated Measures Analyses of Variance and Covariance, first as over-all tests within each strain, and subsequently for each group separately or between groups. Significant responses and groups differences were subsequently located using one- and two-sample Student’s *t*-tests, respectively, at specific times. The plasma catecholamine concentrations, the cardiovascular baselines, and the effect of pre-treatment were first analyzed using one-way ANOVA, and group differences were subsequently located by two-sample Student’s *t*-tests or, in the presence of out-liers, non-parametric Kruskal–Wallis tests. For all analyses, testing proceeded only when significant responses, differences and/or interactions were indicated. The *P*-value was for all tests and each step adjusted according to Bonferroni, except for the catecholamine data, where *P* ≤ 0.05 was considered significant.

## Results

### α_2_AR- and AT_1_R-influence on the plasma catecholamine concentrations

#### Norepinephrine

Similar to that previously described (Berg and Jensen, 2013), the non-selective α_2_AR-antagonist L-659,066 increased the tyramine-induced norepinephrine overflow to plasma in WKY (*P* = 0.015) (Table 2). A similar increase was not seen in SHR, where the plasma norepinephrine concentration was already elevated (*P* < 0.001, WKY compared to SHR controls). Pre-treatment with α_2_AR-agonist alone, i.e., fadolmidine (α_2C>B>A_), ST-91 (α_2(non-A)_), or *m*-nitrobiphenyline (α_2C_) had no effect on overflow in either strain, except for an increase after ST-91 in WKY. After L-659,066 + agonist + tyramine, norepinephrine overflow was not different from that after L-659,066 + tyramine in WKY (*P* = NS), but was much higher in SHR (*P* ≤ 0.025–0.004), also when compared to the SHR PBS + tyramine or corresponding PBS + agonist + tyramine groups (*P* ≤ 0.004).

**Table 2:** **The plasma concentration of norepinephrine and epinephrine at the end of the tyramine-infusion period**.

	WKY			SHR		
	
	
	*N*	Norepinephrine (nM)	Epinephrine (nM)	*N*	Norepinephrine (nM)	Epinephrine (nM)
PBS + tyramine	17	20.6 ± 0.7	2.0 ± 0.9	16	27.4 ± 1.8*	5.0 ± 0.6*
L-659,066_(non-selective)_ + PBS + tyramine	6	26.3 ± 2.0^†^	7.0 ± 1.7^†^	7	30.3 ± 3.4	10.6 ± 2.7
PBS + fadolmidine _(_α_2C>B>A)_ + tyramine	6	18.1 ± 1.3	7.4 ± 1.3^†^	6	23.9 ± 2.2	13.0 ± 2.2^†^
L-659,066 + fadolmidine + tyramine	5	26.6 ± 0.4^†‡^	12.8 ± 1.1^†‡^	6	70.1 ± 16.9^†‡§^	74.8 ± 20.7^†‡§^
PBS + ST-91_(_α_2non-A)_ + tyramine	6	26.5 ± 2.9^†^	5.5 ± 1.8^†^	8	24.0 ± 1.8	11.0 ± 4.1
L-659,066 + ST-91 + tyramine	6	25.4 ± 2.1^†^	12.7 ± 4.2^†^	7	58.3 ± 5.2^†‡§^	49.3 ± 8.0^†‡§^
PBS + *m*-nitrobiphenyline _(_α_2C)_ + tyramine	5	24.1 ± 1.7	4.6 ± 1.5	7	27.9 ± 2.2	8.5 ± 1.6
L-659,066 + *m*-nitrobiphenyline + tyramine	5	24.3 ± 2.0	15.8 ± 4.2^†^	7	50.1 ± 6.0^†‡§^	45.5 ± 15.0^†‡§^
Losartan + tyramine	9	18.4 ± 0.7	4.2 ± 1.5	6	28.4 ± 3.4	11.8 ± 4.1
Losartan + L-659,066 + tyramine	7	26.3 ± 1.9^†||^	25.9 ± 10.4^†||^	7	71.3 ± 10.1^†||§^	41.2 ± 9.3^†||§^
Losartan + clonidine + tyramine	7	17.7 ± 1.1^†^	1.1 ± 0.4^†^	6	19.7 ± 1.1^†||^	1.6 ± 0.8^†||^
Losartan + ST-91 + tyramine		Not done		7	27.4 ± 1.5	15.2 ± 4.4

Differences were detected as indicated between corresponding SHR and WKY control groups (*), between the PBS + tyramine controls and corresponding experimental groups (†), between groups pre-treated with agonist alone (fadolmidine, ST-91, or *m*-nitrobiphenyline) and L-659,066 + the same agonist (‡), between groups pre-treated with losartan alone and losartan + L-659,066/clonidine/ST-91 (||), and between groups pre-treated with L-659,066 alone and L-659,066 combined with agonist or losartan (§). *N*, number of rats per group. *, †, ‡, ||, § - *P* ≤ 0.05.

Losartan alone had no effect on the tyramine-induced norepinephrine overflow in either strain (*P* = NS compared to the controls). Losartan also did not influence the augmenting effect of L-659,066 in WKY (*P* = NS compared to the L-659,066 + tyramine group, and *P* = 0.001 compared to the WKY PBS + tyramine and losartan + tyramine groups). However, in SHR, losartan allowed L-659,066 to greatly increase norepinephrine overflow (*P* ≤ 0.005 compared to PBS/L-659,066/losartan + tyramine groups). Pre-treatment with losartan + clonidine reduced the tyramine-induced norepinephrine overflow in SHR (*P* ≤ 0.048 compared to the PBS/losartan + tyramine groups), and was lower than that in the controls, although not different from that in the losartan + tyramine group, in WKY. Norepinephrine overflow after pre-treatment with losartan + ST-91 was not different from that in the PBS + tyramine or losartan + tyramine groups (tested in SHR only).

#### Epinephrine

The effect of α_2_AR-agonists and antagonist on the surgery-activated epinephrine secretion mostly paralleled their effect on the tyramine-induced norepinephrine overflow in both strains. However, pre-treatment with fadolmidine in both strains, and L-659,066 + *m*-nitrobiphenyline in WKY, increased circulating epinephrine without altering the concentration of norepinephrine.

### The cardiovascular responses

#### The α_2_AR- and AT_1_R-influence on the cardiovascular baselines

**Figure 2:**
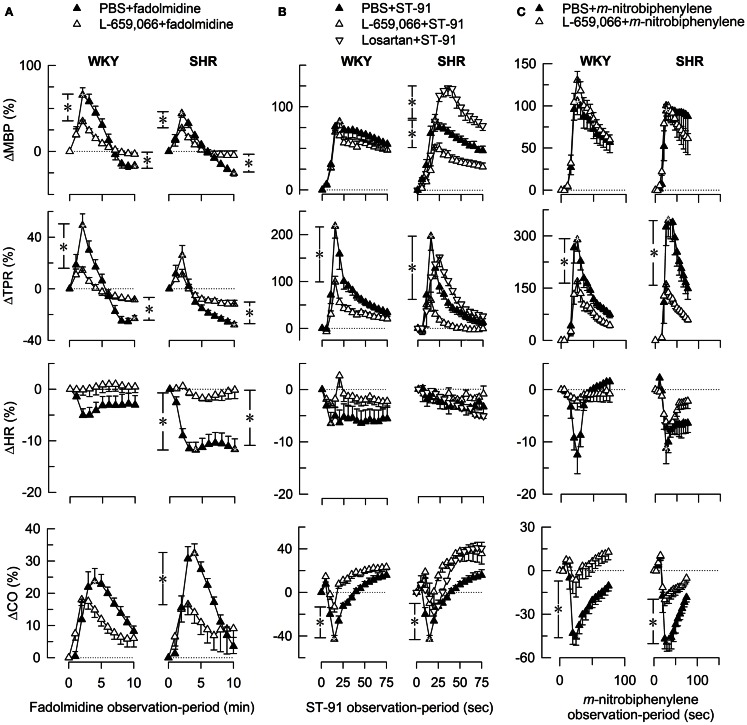
**The MBP-, TPR-, HR-, and CO-response to α2AR-agonists**. Fadolmidine (α_2C>B>A_AR) **(A)**, ST-91 [α_2(non-A)_AR] **(B)**, and *m*-nitrobiphenyline (α_2C_AR, with additional α_2A+B_AR-antagonistic activity) **(C)** were injected alone or after pre-treatment with the non-selective α_2_AR-antagonist L-659,066. The response-curves were analyzed using Repeated Measures Analyses of Variance and Covariance (please see Materials and Methods for details). Significant responses (*within symbols) and group differences (*in brackets) were located as indicated at peak response (all agonists) (brackets left of curves) and after 15 min (fadolmidine only) (brackets right of curves). *, *P ≤ 0.025 for **(A)**, and ≤0.05 for **(B,C)** after curve evaluations.

L-659,066 reduced baseline MBP and TPR in both strains (Table 3). All α_2_AR-agonists induced a transient rise in MBP and TPR (Figure 2, the response to clonidine was similar to that previously published, Berg et al., 2012). Pre-treatment with L-659,066 reduced these TPR-responses, except that of fadolmidine in SHR (Figure 2A), although the MBP-responses were not necessarily reduced. Only fadolmidine subsequently induced an L-659,066-sensitive reduction in MBP and TPR to below baseline in both strains, and also HR in SHR. The agonists had otherwise little effect on baseline HR. Losartan reduced baseline MBP in both strains, HR in WKY, and TPR in SHR (Table 3). Losartan + L-659,066 induced a significant reduction in both HR and TPR in both strains. Losartan increased the MBP-response to ST-91 (Figure 2B) and also the transient rise in CO and MBP in response to clonidine in SHR but had no effect on the HR- or TPR-response to clonidine in either strain (not shown).

**Table 3:** **Cardiovascular baselines prior to tyramine and, in parenthesis, the response to pre-treatment**.

Pre-treatment	WKY					SHR				
	
	
	*N*	MBP (mm Hg)	HR (beats/min)	CO (ml/min)	TPR (mm Hg/ml/min)	*N*	MBP (mm Hg)	HR (beats/min)	CO (ml/min)	TPR (mm Hg/ml/min)
PBS (pooled data)	27	69 ± 3	340 ± 5	32 ± 1	2.2 ± 0.1	26	94 ± 4*	381 ± 6*	19 ± 1*	5.2 ± 0.2*
		(−1 ± 2)	(−5 ± 3)	(2 ± 0)	(−0.3 ± 0.1)		(−3 ± 5)	(−17 ± 4)	(1 ± 1)	(−0.4 ± 0.2)
L-659,066 + PBS	6	62 ± 9^†^	338 ± 13	33 ± 2	1.8 ± 0.2	7	68 ± 6^†^	408 ± 8	18 ± 2	3.9 ± 0.4^†^
		(−16 ± 2)^†^	(−17 ± 9)	(1 ± 1)	(−0.6 ± 0.1)^†^		(−21 ± 4)	(−8 ± 9)	(−1 ± 1)	(−1.1 ± 0.2)
PBS + fadolmidine	6	70 ± 3	346 ± 7	35 ± 3	2.1 ± 0.2	7	73 ± 7^†^	352 ± 10^†^	18 ± 1	4.1 ± 0.2^†^
		(−13 ± 5)	(−12 ± 6)	(4 ± 0)	(−0.8 ± 0.2)		(−27 ± 5)^†^	(−60 ± 10)^†^	(1 ± 1)	(−1.8 ± 0.2)^†^
L-659,066 +	5	50 ± 2^†^	333 ± 11	33 ± 2	1.5 ± 0.1^†^	6	65 ± 7	401 ± 10	18 ± 1	3.7 ± 0.4
fadolmidine		(−23 ± 3)^†^	(−18 ± 9)	(3 ± 1)	(−0.9 ± 0.2)^†^		(−36 ± 10)^†^	(−17 ± 13)	(0 ± 1)	(−1.9 ± 0.4)^†^
PBS + ST-91	6	82 ± 5	349 ± 4	33 ± 5	2.8 ± 0.5	8	82 ± 4	378 ± 13	14 ± 1	6.1 ± 0.6
		(−6 ± 2)	(−30 ± 6)	(5 ± 1)	(−0.7 ± 0.0)^†^		(−14 ± 6)	(−35 ± 9)	(0 ± 1)	(−1.2 ± 0.4)
L-659,066 + ST-91	6	67 ± 6	345 ± 7	33 ± 2	2.0 ± 0.1	7	79 ± 10	412 ± 14	15 ± 2	6.4 ± 1.
		(−4 ± 5)	(−10 ± 9)	(8 ± 2)	(−0.9 ± 0.2)^†^		(−26 ± 7)	(−22 ± 8)	(0 ± 1)	(−1. ± 0.4)
PBS +	5	85 ± 1	390 ± 25	31 ± 3	2. ± 0.2	7	137 ± 8	386 ± 6	18 ± 1	7.5 ± 0.5^†^
*m*-nitrobiphenyline		(−9 ± 5)	(−4 ± 11)	(2 ± 1)	(−0.5 ± 0.2)		(39 ± 8)	(−10 ± 6)	(1 ± 2)	(1.8 ± 0.8)
L-659,066 +	7	59 ± 2	349 ± 10	30 ± 4	1.9 ± 0.2	7	115 ± 8	420 ± 8	20 ± 1	5.8 ± 0.5
*m*-nitrobiphenyline		(−11 ± 3)	(−12 ± 6)	(1 ± 2)	(−0.5 ± 0.1)		(19 ± 7)	(8 ± 7)	(1 ± 1)	(0.8 ± 0.4)
Losartan	9	53 ± 3	341 ± 12	31 ± 2	1.8 ± 0.1	6	72 ± 5^†^	376 ± 7	13 ± 1^†^	5.5 ± 0.3
		(−22 ± 4)^†^	(−25 ± 5)^†^	(0 ± 2)	(−0.7 ± 0.2)		(−24 ± 8)^†^	(−19 ± 6)	(−3 ± 1)	(−1.0 ± 1.0)^†^
Losartan + L-659,066	9	38 ± 3^†‡^	310 ± 7^†^	22 ± 2^†‡^	1.8 ± 0.1	7	41 ± 3^†‡^	348 ± 17^†^	10 ± 2^†^	5.2 ± 1.0
		(−23 ± 2)^†^	(−44 ± 8)^†^	(−2 ± 1)	(−0.9 ± 0.2)^†^		(−48 ± 8)^†^	(−73 ± 9)^†‡^	(−6 ± 2)^†^	(−0.6 ± 0.8)
Clonidine	7	65 ± 3	314 ± 7	40 ± 3	1.6 ± 0.1^†^	6	60 ± 4^†^	320 ± 11^†^	18 ± 1	3.4 ± 0.2^†^
		(−4 ± 6)	(−33 ± 8)	(11 ± 1)^†^	(−0.8 ± 0.2)^†^		(−35 ± 10)^†^	(−121 ± 19)^†^	(2 ± 1)	(−2.5 ± 0.6)^†^
Losartan + clonidine	6	54 ± 3	346 ± 7	35 ± 3	1.5 ± 0.1^†^	6	44 ± 3^†‡^	314 ± 8^†‡^	13 ± 3	4.2 ± 1.0
		(−22 ± 7)	(−34 ± 13)	(10 ± 2)^†‡^	(−1.4 ± 0.2)^†‡^		(−65 ± 9)^†‡^	(−134 ± 12)^†‡^	(−2 ± 2)	(−2.9 ± 1.3)^†^
Losartan + ST-91			Not done			7	54 ± 4^†‡^	358 ± 11	13 ± 1^†^	4.7 ± 0.6
							(−50 ± 7)^†^	(−69 ± 8)^†‡^	(−5 ± 1)^†^	(−1.5 ± 0.3)

Cardiovascular baselines in the PBS-control groups are shown as pooled data from experiments run at different times. However, statistical evaluation of the effect of pre-treatment was done using control rats from the same set of experiments. Comparisons were made between the WKY and SHR controls (*), between the PBS-controls and the experimental groups (†), between the groups pre-treated with PBS + agonist (fadolmidine, ST-91, or nitrobiphenyline) and corresponding groups given L-659,066 + the same antagonist (significant differences not detected), and between the groups pre-treated with losartan alone and losartan + L-659,066/clonidine/ST-91 (‡).*P ≤ 0.0125, †, ‡ *P* ≤ Bonferroni adjusted *P*-value for each set of experiment.

#### The α_2_AR- and AT_1_R-influence on the cardiovascular response to tyramine

**Figure 3:**
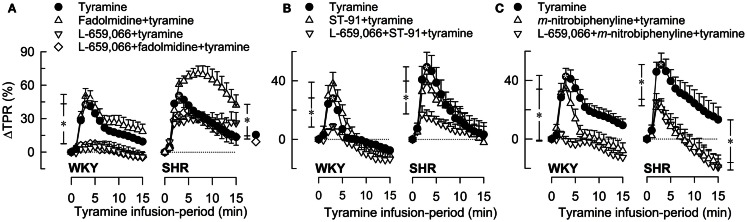
**The TPR-response to tyramine-induced norepinephrine release after pre-treatment with α_2(non-A)_AR-selective agonist, alone or combined with L-659,066**. The peripherally restricted α_2C>B>A_AR-agonist fadolmidine **(A)**, the peripherally restricted α_2(non-A)_AR-selective agonist ST-91 **(B)**, and the α_2C_-selective agonist *m*-nitrobiphenyline with additional α_2A+B_AR-antagonistic activity **(C)** were injected alone or after pre-treatment with the peripherally restricted α_2_AR-antagonist L-659,066. Baselines prior to tyramine are shown in Table 3. Significant responses (*within symbol) and differences between the control and experimental groups were located at peak response (*brackets left of curves) and at 15 min (*brackets right of curves). *, *P ≤ 0.025 after curve evaluations.

As previously documented (Berg et al., 2010; Berg and Jensen, 2013), tyramine induced an immediate, but transient rise in TPR (Figure 3) and a sustained increase in MBP, HR, and CO. The present results focused on the effect of pre-treatment on the TPR-response to tyramine, and the concomitant changes in MBP, HR, and CO (all expressed in % of baselines) are therefore shortly described but not shown.

Pre-treatment with α_2_AR-agonist alone (Figures 3A–C), i.e., fadolmidine, ST-91, or *m*-nitrobiphenyline, had no effect on the TPR-response to tyramine in WKY (*P* = NS). In SHR, the TPR-response to tyramine was increased after fadolmidine (*P* = 0.023 at 15 min), not influenced by ST-91, and decreased after *m*-nitrobiphenyline (*P* = 0.003 at 3 min). L-659,066 alone (Figure 3A) virtually eliminated the TPR-response in WKY (*P* ≤ 0.008), with no additional effect when combined with agonist (Figures 3A–C). In SHR, L-659,066 alone did not change the tyramine-induced rise in TPR, but abolished the response when combined with ST-91 (Figures 3A,B). The response to tyramine in L-659,066 + fadolmidine-pre-treated SHR was less than that after fadolmidine alone, although not different from that in the controls (Figure 3A). Moreover, ΔTPR was not further reduced after L-659,066 + *m*-nitrobiphenyline compared to that after *m*-nitrobiphenyline alone in SHR (Figure 3C).

A reduced MBP-response to tyramine after L-659,066, alone or combined with agonist (fadolmidine, ST-91, or *m*-nitrobiphenyline), was observed in WKY, but only after L-659,066 + agonist in SHR. *m*-Nitrobiphenyline alone reduced ΔMBP in both strains. The agonists had little effect on the tyramine-induced tachycardia, except fadolmidine which increased ΔHR in SHR. A lower tyramine-induced rise in CO was observed after fadolmidine and ST-91 in WKY, after fadolmidine in SHR, and in all groups given L-659,066 as part of the pre-treatment.

**Figure 4:**
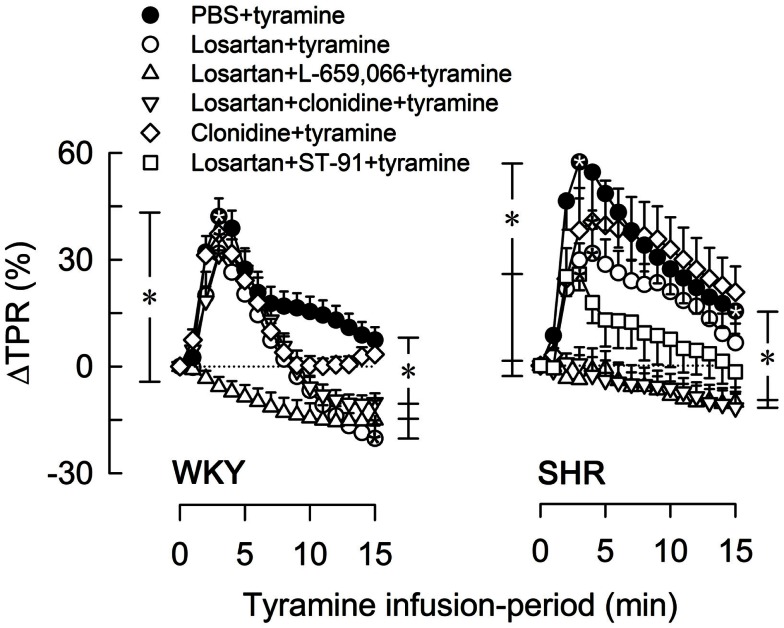
**The TPR-response to tyramine after pre-treatment with the AT_1_R-antagonist losartan, alone or combined with L-659,066, clonidine, or ST-91**. The effect of losartan + ST-91 was tested in SHR only. Baselines prior to tyramine are shown in Table 3. Significant responses (*within symbol) and group differences were detected at peak response (*brackets left of curves) and at 15 min (*brackets right of curves) as indicated. *, *P ≤ 0.025 after curve evaluations.

Losartan alone had no effect on the TPR-peak response to tyramine in either strain, but induced a vasodilatory TPR-response at the end of the tyramine-infusion in WKY (Figure 4). Like L-659,066 alone (Figure 3A), losartan + L-659,066 eliminated the TPR-peak response to tyramine in WKY (Figure 4), and in addition caused a fall in TPR to below baseline. Losartan + clonidine, like clonidine alone, had no effect on the TPR-response to tyramine in WKY (Figure 4). In SHR, losartan + L-659,066 and losartan + clonidine, unlike losartan, L-659,066 or clonidine alone, eliminated the TPR-response to tyramine. The TPR-peak response was reduced also after pre-treatment with losartan + ST-91 (tested in SHR only, Figure 4). Losartan did not alter the MBP-response to tyramine, but increased the CO-response in both strains. This increase was eliminated when losartan was combined with L-659,066, and in WKY also with clonidine. The tyramine-induced tachycardia was increased in SHR after losartan + clonidine, similar to that seen after clonidine alone.

## Discussion

The main finding in the present study was that the failing α_2A_AR inhibition of peripheral norepinephrine and epinephrine release in SHR during tyramine-stimulated norepinephrine release was restored by stimulation of the α_2C_AR or inhibition of the AT_1_R. α_2C_AR-stimulation and AT_1_R-inhibition also restored the failing postsynaptic α_2_AR control of vascular tension in SHR.

As previously described (Berg and Jensen, 2013), α_2_AR-mediated auto-inhibition of peripheral catecholamine release was demonstrated in tyramine-stimulated WKY by an increased norepinephrine overflow to plasma after pre-treatment with the non-selective α_2_AR-antagonist L-659,066. This increase was eliminated after addition of the non-selective α_2_AR-agonist clonidine (Berg and Jensen, 2013), but not, as demonstrated by the present experiment, by agonists with less or no α_2A_AR reactivity, such as fadolmidine, ST-91, or *m*-nitrobiphenyline. Clonidine reduced the tyramine-induced norepinephrine overflow in SHR, and this reduction was fully reversed by L-659,066 (Berg and Jensen, 2013), and, again, a similar decrease was not seen after fadolmidine, ST-91, or *m*-nitrobiphenyline. Both tyramine and L-659,066 are peripherally restricted, i.e., do not pass the blood-brain barrier (Oldendorf, 1971; Clineschmidt et al., 1988). Inhibition of tyramine-stimulated norepinephrine overflow therefore involved in both strains peripherally located α_2_AR, predominantly of the α_2A_-subtype, in agreement with that previously observed by others (Starke, 2001; Brede et al., 2004).

Epinephrine is secreted directly into blood and not subjected to local re-uptake, and release is therefore not stimulated by tyramine (Berg and Jensen, 2013). However, the stress induced by the surgical procedure activated some secretion of epinephrine from the adrenals (Berg et al., 2012). Clonidine precipitated an L-659,066-sensitive reduction in this secretion in both strains (Berg et al., 2012; Berg and Jensen, 2013), whereas fadolmidine, ST-91, or *m*-nitrobiphenyline did not. It therefore appeared that the α_2A_AR inhibited also the secretion of epinephrine, in agreement with previous studies on the rat adrenal gland (Lymperopoulos et al., 2007). This differed from that in the mouse, where the α_2C_-subtype inhibited epinephrine secretion (Brede et al., 2003; Moura et al., 2006).

Although clonidine reduced tyramine-induced norepinephrine overflow to plasma in SHR, the antagonist L-659,066 failed to increase overflow in this strain (Berg and Jensen, 2013). This malfunction depended on the tyramine-stimulated release of norepinephrine, since L-659,066, and also the α_2_AR-antagonist yohimbine, clearly increased norepinephrine overflow in SHR not stimulated with tyramine but where NET-re-up-take was blocked by desipramine (Berg et al., 2012). However, norepinephrine overflow was greatly increased in tyramine-stimulated SHR when L-659,066 was combined with the α_2C_AR-reactive agonist fadolmidine, which has a 35 and 10 times higher affinity for the α_2C_- and α_2B_AR than the rat α_2A_-subtype, respectively (Lehtimaki et al., 2008). Overflow was also greatly increased when L-659,066 was combined with the non-A-selective ST-91 (Takano et al., 1992), or the α_2C_AR-selective *m*-nitrobiphenyline, which in addition has an α_2A+B_AR-antagonistic effect (Crassous et al., 2007). Since fadolmidine and ST-91 do not cross the blood-brain barrier (Clineschmidt et al., 1988; Lehtimaki et al., 2008), stimulation of peripheral α_2C_AR appeared to re-establish α_2A_-auto-inhibition in SHR (Figure 1).

Augmented tyramine-induced norepinephrine overflow was also observed in SHR but not in WKY after pre-treatment with losartan + L-659,066, whereas losartan alone had no effect. G-protein G_q_-signaling agents, including angiotensin II through the AT_1_R, have been shown in isolated mouse atria to stimulate norepinephrine release by interfering with down-stream signaling of the inhibitory α_2_AR-G_i_ pathway (Figure 1) (Cox et al., 2000; Trendelenburg et al., 2003a). The AT_1_R interaction involved only the α_2C_- and not the α_2A_-subtype (Cox et al., 2000; Trendelenburg et al., 2003a). α_2C_AR-agonist may therefore restore α_2A_-auto-inhibition by counter-acting the AT_1_R-G_q_-interference, and losartan by eliminating the AT_1_R-interference. Thus, as could be expected, ST-91 did not alter the tyramine-induced norepinephrine overflow after losartan in SHR. The present results were therefore compatible with studies showing that the reduced afferent renal nerve signaling observed in response to efferent renal sympathetic nerve activation was increased in SHR by the α_2_AR-antagonist rauwolscine, and further potentiated when rauwolscine was combined with losartan, whereas losartan alone had no effect (Kopp et al., 2011).

However, the experimental approach is indirect and performed in the whole animal, and other explanations should therefore also be considered. For instance, α_2C_AR-stimulation will hamper renal renin release (Michel and Rump, 1996), and, through that, may lower AT_1_R-activation and stimulation of release. However, if this was the mechanism responsible, one might have expected losartan alone to lower the release of norepinephrine, which it did not. Unlike vesicular release, NET-mediated release has been considered not to be regulated by presynaptic receptors (Starke, 2001). However, recent studies show that NET may indeed be influenced by presynaptic control, as demonstrated by the hampering effect of muscarinic receptor activation on the NET transport rate (Parker et al., 2010), a response which in other cells is mediated through a PKC-dependent pathway (Apparsundaram et al., 1998). However, PKC did not seem to influence tyramine-induced transport through NET, since preliminary studies showed that the PKC-inhibitor staurosporine, like losartan alone, did alter norepinephrine overflow (plasma norepinephrine concentration = 19.8 ± 2.3 and 27.1 ± 2.3 nM in WKY and SHR, respectively, five rats/group, *P* = NS compared to the controls, Berg, unpublished observations). α_2_AR-agonists have also been shown to bind to NET and to competitively inhibit re-uptake of a norepinephrine analog (Park et al., 2013). This response was not prevented by α_2_AR-antagonist, and was therefore likely to result from their structural similarity to norepinephrine and not from α_2_AR-signaling. Agonist inhibition of NET did not seem to alter the tyramine-induced reversed transport of norepinephrine through NET, since none of the present agonists lowered tyramine-induced overflow, and the reduction observed in SHR after clonidine was abolished by L-659,066 (Berg and Jensen, 2013).

The secretion of epinephrine mostly followed the same pattern as that of norepinephrine overflow, indicating that α_2A_AR failed to inhibit also epinephrine secretion in SHR, and that this malfunction could be restored by α_2C_AR-stimulation or AT_1_R-inhibition.

The tyramine-stimulated norepinephrine overflow after L-659,066 + agonist and losartan + L-659,066 was about two times greater, and that of epinephrine 10 times greater, than that in the control or L-659,066-only groups in SHR, but not higher than that after pre-treatment with L-659,066 alone in WKY, i.e., 28% higher than in the controls. L-659,066 and yohimbine greatly increased the plasma concentration of norepinephrine and epinephrine also in desipramine-treated, non-stimulated SHR (Berg et al., 2012). These observations suggested an up-regulation of peripheral, presynaptic α_2A_AR in SHR, in order to down-regulate the elevated sympathetic tone and/or to compensate for the failing α_2_AR-auto-inhibition in this strain.

L-659,066 reduced baseline MBP and TPR in both strains, but abolished the tyramine-induced rise in TPR in WKY only. Also the G_i_-inhibitor pertussis toxin eliminated the TPR-response to tyramine in this strain alone (Berg et al., 2009). The abolished TPR-response was most likely due to that L-659,066 inhibited postsynaptic, VSMC α_2_AR-G_i_-signaling, thereby allowing VSMC βAR-adenylyl cyclase-mediated dilatation to oppose the norepinephrine-induced, α_1_AR-mediated vasoconstriction. Also this α_2_AR-function failed in SHR. The malfunction appeared to be precipitated by the stimulated release of norepinephrine, since a strain-related difference was not seen in the moderating effect of L-659,066 on the TPR-response to exogenous α_1_AR-agonist (Berg et al., 2012). Like the failing control of catecholamine release, also this disorder was repaired by AT_1_R-inhibition or α_2C_AR-stimulation, since losartan + L-659,066 and L-659,066 + ST-91 eliminated the TPR-response to tyramine. This may be due to the high norepinephrine and/or epinephrine release in these SHR groups, which, in the presence of the α_2_AR-antagonist inhibiting VSMC α_2_AR, may be sufficient to re-establish a βAR-mediated counter-action of the norepinephrine-induced α_1_AR-mediated vasoconstriction. This conclusion is in agreement with our previous study showing that neuronally activated, β_1_AR-mediated vasodilatation counter-acted the TPR-response to tyramine in WKY only, whereas β_2+3_AR activated by epinephrine from the adrenals opposed the late half of the TPR-response in SHR (Berg et al., 2010). The TPR-response to tyramine in SHR was also eliminated after losartan + clonidine and reduced after losartan + ST-91, in spite of a normal plasma norepinephrine concentration. It is therefore possible that also the failing β_1_AR contribution to TPR-control in SHR resulted from VSMC AT_1_R-activation.

In agreement with studies on genetically modified mice, where the initial clonidine-induced vasoconstriction was due to activation of VSMC α_2B_AR (Link et al., 1996), the present agonists, and as previously described also clonidine (Berg et al., 2012), induced a transient rise in TPR, which was reduced or eliminated by L-659,066, except that of fadolmidine in SHR. The L-659,066-sensitive fraction of this vasoconstriction may be mediated through the α_2B_AR on VSMC, although the present experiments could not exclude a role of the α_2A_AR. However, the L-659,066-sensitive fraction of the response to *m*-nitrobiphenyline was likely to be mediated through VSMC α_2C_AR, since this α_2C_-selective agonist also acted as an α_2A+B_AR-antagonist (Crassous et al., 2007). Although VSMC α_2C_AR did not contribute to BP control in genetically modified mice (MacDonald et al., 1997), stimulated α_2C_AR-mediated vasoconstriction has been demonstrated in veins and arterioles (Chotani et al., 2004; Görnemann et al., 2007). The L-659,066-insensitive part of the agonist-induced vasoconstriction was likely to be mediated through α_1_AR, since at least fadolmidine contained α_1_AR-agonistic activity (Lehtimaki et al., 2008). The latter component may also explain why fadolmidine increased the TPR-response to tyramine in SHR. This increase was absent after additional pre-treatment with L-659,066, possibly due to that L-659,066, by inhibiting the VSMC α_2_AR-G_i_ pathway, allowed norepinephrine-stimulated, βAR-mediated vasodilatation, in that manner opposing the tyramine-induced, α_1_AR-mediated vasoconstriction. Fadolmidine was the only agonist which induced a late L-659,066-sensitive fall in MBP, TPR, and HR in SHR, possibly due to its α_2A_AR-component, which may lower catecholamine release prior to tyramine-stimulation and/or stimulate endothelial, vasodilatory α_2A_AR (Shafaroudi et al., 2005). The TPR-response to tyramine was reduced by *m*-nitrobiphenyline. This reduction was not further influenced by additional pre-treatment with L-659,066, and was therefore likely to result from the α_2A+B_AR-antagonistic effect of this agonist. The TPR-response was therefore more sensitive to the promiscuity of the α_2_AR-agonists than the α_2_AR-mediated control of catecholamine release.

## Conclusion

Peripheral α_2_AR represent the last line of defense against adrenergic hyperactivity. The α_2A_-subtype played a dominating role in limiting peripheral catecholamine release in WKY, but failed to do so in SHR. This malfunction was restored after α_2C_AR-stimulation or AT_1_R-inhibition, suggesting that an AT_1_R-G_q_/α_2C_AR-G_i_-interaction disturbed normal α_2A_AR-mediated control of catecholamine release in SHR. This α_2C_AR-AT_1_R-interaction may be responsible for the elevated plasma norepinephrine concentrations observed in SHR, and contribute to the sympathetic hyperactivity and hypertension in this strain. A loss-of-function α_2C_AR deletion polymorphism has been shown to be more frequent in African–Americans and connected to a greater HR- and BP-response in the cold-pressor-test (Kurnik et al., 2008). An augmented sympathetic response to this stress-test is linked to increased cardiovascular morbidity (Matthews et al., 2004), and heart failure patients with the same α_2C_AR polymorphism had a worsened prognosis and increased risk of heart failure (Small et al., 2002, 2003). Estrogen stimulated the expression of α_2C_AR in human dermal arteriole VSMC (Eid et al., 2007), and may from the present results provide a mechanism whereby estrogen protects against hypertension. A failing α_2A_AR auto-inhibition of catecholamine release due to an AT_1_R-α_2C_AR interaction may therefore be highly relevant for development of hypertension, the major risk factor for cardiovascular events.

## Conflict of Interest Statement

The authors declare that the research was conducted in the absence of any commercial or financial relationships that could be construed as a potential conflict of interest.
